# Research on Methods Decreasing Pesticide Waste Based on Plant Protection Unmanned Aerial Vehicles: A Review

**DOI:** 10.3389/fpls.2022.811256

**Published:** 2022-07-07

**Authors:** Heming Hu, Yutaka Kaizu, Jingjing Huang, Kenichi Furuhashi, Hongduo Zhang, Ming Li, Kenji Imou

**Affiliations:** ^1^Graduate School of Agricultural and Life Sciences, The University of Tokyo, Tokyo, Japan; ^2^Hunan Agricultural Equipment Research Institute, Changsha, China; ^3^Hunan Advanced Engineering Technology Research Center for Agricultural Aviation Hunan, Changsha, China

**Keywords:** unmanned aerial vehicle, precision agriculture, droplet drift, droplet deposition, electrostatic spray, variable spray

## Abstract

In plant protection, the increasing maturity of unmanned aerial vehicle (UAV) technology has greatly increased efficiency. UAVs can adapt to multiple terrains and do not require specific take-off platforms. They do well, especially in farmland areas with rugged terrain. However, due to the complex flow field at the bottom of a UAV, some of the droplets will not reach the surface of a plant, which causes pesticide waste and environmental pollution. Droplet deposition is a good indicator of the utilization rate of pesticides; therefore, this review describes recent studies on droplet deposition for further method improvement. First, this review introduces the flight altitude, speed, and environmental factors that affect pesticide utilization efficiency and then summarizes methods to improve pesticide utilization efficiency from three aspects: nozzles, electrostatic sprays, and variable spray systems. We also point out the possible direction of algorithm development for a UAV’s precision spray.

## Introduction

Global food production has been threatened due to pests, weeds and diseases (Savary et al., 2019; Zhang et al., 2021). Currently, the main control method uses chemicals. With the increasing maturity of UAVs technology, the proportion of UAV spraying in the overall improvement of chemical control methods has increased significantly (Zhang et al., 2021). Compared with traditional plant protection machinery, UAVs have the advantages of flexible operation and adaptation to various terrains from paddy fields to hilly areas. In some parts of Asia, such as Japan, Southeast Asia, and southern China, due to the rugged terrain and scattered fields, large machinery cannot enter. UAV spraying is a good solution to this problem. UAV has the characteristics of taking off and landing anywhere and high work efficiency. The workload of a day exceeds 300 acres.

**FIGURE 1:**
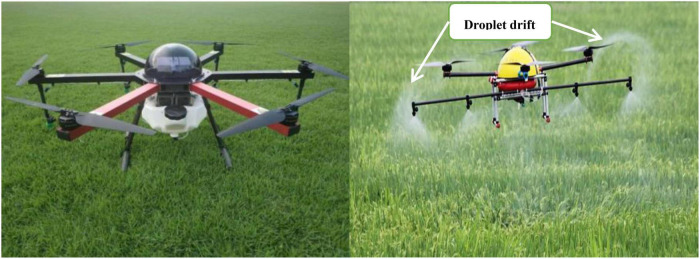
Plant protection UAV and droplet drift phenomenon.

However, pesticides sprayed from the sprayer of the UAV often drifted (Wang Z. et al., 2020) and evaporated (Wang G. et al., 2020). Drifted pesticide would flow into the soil or rivers, and the evaporated pesticide would eventually flow into soil and rivers with rainfall, causing environmental pollution (Pérez-Lucas et al., 2019) and increasing pest resistance (Jiang et al., 2012). Figure 1 shows the latest type of plant protection UAV and the phenomenon of droplet drift, Typically, some pesticide droplets were thrown into the air by air currents, evaporated before reaching the ground in high temperatures, follow the rainfall into rivers or land to cause environmental pollution (Kumar et al., 2018). Finally only deposited droplets would control pests and weed, therefore, understanding the movement of droplets, reducing the evaporation and drift of pesticide droplets, and enhancing the droplet deposition amount can become the direction of researchers’ efforts.

Mainly, studies on decreasing pesticide droplets waste on UAV have been based on Computational Fluid Dynamics (CFD) simulation tests, wind tunnel tests (Fritz et al., 2013), and field tests. CFD technology used the simulation environment provided by the computer to analyze the movement and deposition of droplets under different environmental wind speeds, different flight speeds and altitudes. Similarly, the wind tunnel test simulates the movement of droplets under different wind speeds through an artificial wind field. The main indicators to measure the effect of droplet deposition were deposition amount and deposition density (Guo et al., 2019). The deposition amount is the quality of the droplet deposition per unit area, and the deposition density is the number of droplets per unit area. These two parameters will be used as the main factors to measure the efficiency of the spray system.

This review introduces UAV operation parameters that may affect the deposition of droplets, as well as techniques to improve the effect of droplet deposition, and proposes new research ideas based on the summary of previous studies.

## Unmanned Aerial Vehicle Operation Parameters Affecting the Deposition Effect

The factors that affect the droplet deposition effect include flight speed, flight altitude, droplet size, ambient temperature, humidity, and ambient wind speed (Qiu et al., 2013; Al Heidary et al., 2014; Zhu et al., 2019). In UAV plant protection, the flight speed and ambient wind speed are directly related to droplet drift. The mixed wind field generated by the two will change the original trajectory of the droplet. Humidity and temperature are directly related to the evaporation of droplets, and then the flight height is related to evaporation and droplet drift. Because of the different flight heights, the time for droplets to fall will be different, and the probability of evaporation in the air is also different, the different flying heights, the droplets are affected by the environmental wind field when they fall, which will also affect the drifting distance. Some of these factors can be adjusted during UAV flight operations to obtain the optimal droplet deposition effect. Methods to test the effect of droplet deposition are CFD simulation analysis and the water-sensitive paper detection method (Cunha et al., 2012). The following studies show how researchers approached the optimal UAV operation parameters for spraying work.

### Flight Speed

Flight speed affects the time of the UAV’s stay on the top of crop canopy, which determined the pesticide deposition effect. Therefore, suitable flight speed can improve the working efficiency of UAV while ensuring the amount of droplet deposition, here we counted the droplet deposition effects of different scholars at different speeds, as shown in Table 1. Research on the influence of flight speed on the deposition effect mainly included simulation analysis and outdoor experiments. Lv et al. (2019) investigated indoor simulation experiments on the influence of flight speed on droplet deposition and concluded that a higher flight speed resulted in a lower droplet deposition performance. They observed that when flight speed reached 1 m/s the deposition density decreased to 41.4%, and the coverage decreased to 3.9%. Shi et al. (2019) built up CFD simulation conditions in Ansys Fluent software to investigate the optimal droplet deposition result of the UAV. Specific wind speed, flight speed, and other conditions were simulated, and the movement trajectory of the droplets was obtained, to determine the result of the droplet deposition. Under the conditions of a flight speed of 3 m/s and altitude of 1.5 m, the deposition concentration was 50–200 μg/cm2. It has been in Zhang et al. (2012) that, through a thermal infrared imaging instrument method to compare the difference in deposition results at different flight altitudes and flight speeds, it is found that the best flight speed of the UAV is 1.5 m/s. Pan et al. (2021) tested the impact of diesel-powered UAVs on the deposition effect of litchi trees at different speeds at the same flying altitude. The results showed that the deposition effect is the best when the flying speed is up to 2.8 m/s. An experiment in the rice field indicated that when the UAV flight speed was kept at a low speed (2 m/s), the droplet performance showed better uniformity and higher droplet deposition density, than when flight speed was higher (4 and 6 m/s) (Kharim et al., 2019). The experimental results of Hunter et al. (2020b) show that the spray drift phenomenon is the least when an air-sensing flat spray or a turbine air-sensing flat spray nozzle and an application speed of 3 m/s are applied.

**TABLE 1:** Published research on the effect of flight speed on droplet deposition.

Source	Crop	Methodology	Optimal flight speed (m/s)	Coverage (%)	Density (droplets/cm^2^)	Deposition (μg/cm^2^)
Zhang et al. (2012)	Rice	• Indoor spray test • Infrared imaging	1.5	N/A	N/A	300
Kharim et al. (2019)	Rice	• Outdoor spray test • Water-sensitive paper	2.0	12.0	60.0	N/A
Lv et al. (2019)	N/A	• Indoor spray test • Infrared imaging	0.7	10.5	70.3	N/A
Ahmad et al. (2020)	Weeds	• Outdoor spray test • Water-sensitive paper	2.0	35.0	120.0	N/A
Shi et al. (2019)	N/A	• Simulation analysis	3.0	N/A	N/A	120
Pan et al. (2021)	Litchi tree	• Outdoor spray test	2.8	5.3	53.1	136
Zhou and He (2016)	Tea tree	• Indoor spray test	0.7	8.9	28.1	N/A

In general, at the same altitude, a faster flight speed will cause the worse droplet deposition effect. As the flight speed increases, the uniformity of the droplet distribution is improved, while the droplet density and spray coverage percentage decrease (Zhou and He, 2016). However, to ensure the efficiency of the operation, a balance is often necessary between the flight speed and the deposition effect; normally, the operating flight speed range from 1.5 to 3 m/s.

### Flight Altitude

As is shown in Table 2, the flying altitude of UAVs affects the deposition on different canopies of plants, so the suitable flight altitude for UAVs varies greatly among different plants. In a cotton field, when the UAV’s flying height is 2 m, the deposition of droplets on different canopies is more uniform. When the height is lower than 2 m, the strong downward airflow makes the droplet deposition on different cotton canopies decrease significantly (Lou et al., 2018). The flying altitude of the UAV should be less than 2.5 m (Wang J. et al., 2020) when it is spraying pineapples. When the flight altitude was higher than 2.5 m, the droplet deposition decreased suddenly. Shengde et al. (2017b) executed experiments on the influence of UAV parameters on droplet deposition on an orange tree canopy, and found the optimal droplet deposition density at a flying altitudes of 2.5 m and spray rate of 1 L/min, and the average deposition density was 128.16 droplets/cm^2^. Hou et al. (2018), completed an experiment on the optimal deposition performance on trees. Droplet deposition reached a climax when the flight altitude was 1.2 m, and the deposition performance was much lower when the flight altitude was 0.6 and 1.8 m. Flight altitudes too high or too low were not conducive to the deposition of droplets on the surface of the crop. The flight altitude range for deposition is 1.0–2.5 m, and different UAV or spray systems will have different optimal flight altitudes. Hu et al. (2021) proposed that a flying altitude of 1.5 m and a spray volume of 22.5 L/ha was the best parameters to use for UAV spraying to control aphids in cotton seedlings. Another group used the UAV and Taguchi methods to study the optimal droplet distribution control parameters for citrus trees (Hou et al., 2019). The best results were observed with an inverted triangle citrus canopy shape, a spray height of 1.40 m, and a flight speed of 1.0 m/s. The spraying height has a significant influence on the droplet distribution on the upper layer of wheat. The height of spraying was 5.0 m and the spraying speed was 4 m/s. The coverage rate of droplets on the lower layer of wheat was the largest (Qin et al., 2018).

**TABLE 2:** Published research on the effect of flight altitude on droplet deposition.

Source	Crop	Methodology	Optimal flight speed (m/s)	Coverage (%)	Density (droplet/cm^2^)	Deposition (μg/cm^2^)
Zhang et al. (2015)	N/A	• Numerical analysis	6	N/A	N/A	80
Wang C. et al. (2016)	Wheat	• Outdoor spray test • Water-sensitive papers	3	45.8	N/A	N/A
Chen et al. (2019)	N/A	• Indoor spray test • Orthogonal test	2.5	N/A	114	N/A
Lou et al. (2018)	Cotton	• Outdoor spray test • Kromekote card	2	9.35	N/A	N/A
Hou et al. (2019)	Citrus	• Outdoor spray test	1.2	3.94	18.75	N/A
Hussain et al. (2019)	N/A	• Outdoor spray test	1.5	N/A	150	N/A
Hu et al. (2021)	Cotton	• Outdoor spray test	1.5	9.43	110	N/A

In addition, in outdoor conditions, wind speed and flying altitude usually work together to affect the deposition. Therefore, some researchers have considered both wind speed and altitude at the same time. Wang C. et al. (2016) proposed that under the conditions of a 2.0–3.5 m height, 5.0 ± 0.3 m/s speed and 0.8 m/s, crosswind speed, the higher the height, the better the uniformity of the droplet distribution. Hussain et al. (2019) performed a droplet sedimentation experiment with 50, 75, and 100% nozzle openings under the conditions of 1.5–3.0 m and wind speeds of 1.0–5.8 m/s. The experimental results showed a lower the wind speed and height would be beneficial to the droplet settlement effect. At a height of 2 m, good uniformity is achieved at 50, 75, and 100% nozzle openings in the range of 1.0–3.8 m/s wind speed. Through simulation experiments, Zhang et al. (2015) found that the flying speed was 3 m/s, the crosswind speeds were 1, 2, and 3 m/s, and the flying heights were 5, 6, and 7 m. A turbulence model with an approximate solution to the N-S equation with appropriate boundary conditions was established. Through simulation and experimental results, the following conclusions can be drawn: the crosswind velocity is a greater influencing factor than the spray height in the air is an influencing factor for the droplet drift; and droplet drift only occurs in the spray field downwind.

Therefore, it is necessary to establish a UAV deposition model according to different crop canopies and to consider the influence of wind speed and altitude on deposition during operation.

### Pump Pressure

The most important factor that determines the size of the droplets was the pressure of the water pump. Here we mainly discussed the relationship between particle size and droplet deposition. According to the results of wind tunnel experiments to test droplet deposition with different types of nozzle parameters, a larger droplet size had a better performance on anti-drift and droplet deposition (Wen et al., 2016; Wang C. et al., 2020). Zhang et al. (2018) established a CFD model to study the relationship between droplet size and droplet drift under crosswind conditions. When the droplet size is less than 150 μm, the phenomenon of droplet drift is obvious, and as the particle size becomes larger, the droplet drift phenomenon disappears. However, Wang S. L. et al. (2017) found that when UAVs are used to detect wheat field pest control effects, the size of droplets is negatively correlated with deposition density. Part of the reason is that when the coverage is similar but the particle size is larger, there are fewer droplets per unit area. Qin et al. (2016) completed a paddy field experiment to find the most suitable particle size range for droplet deposition, it was found that the droplet size is very prone to drift when the droplet size is less than 100 μm, while it is difficult to penetrate the canopy layer of crops when droplet size is greater 400 μm, in this case, the deposition effect of droplets is not good. Droplet that are too large or too small are not conducive to their deposition of droplets. The approximate suitable range for droplet deposition is 100–300 μm. According to different crops and external environments, the most suitable range will be adjusted accordingly. A UAV has better deposition and efficacy control with coarse nozzles at higher spray volumes (> 16.8 L ha^–1^), but it has better performance at lower spray volumes (< 9.0 L ha^–1^) and fine nozzles. Poor deposition and efficacy control (Wang et al., 2019a). Fawaz (2018) used a UAV to carry out an aerial spraying experiment with same spray rate and different particle size droplets on the rice canopy. Four TEEJET nozzles with different orifice sizes were used (the volume median diameter (VMD) of these droplets was 95.21, 121.43, 147.28, and 185.09 μm, respectively), and the drift of the droplets in the target area and droplets in the non-target area were determined. The distributions were compared and analyzed. The results showed that as the droplet size increased, the droplet deposition rate on the upper and lower rice canopies of the target area increased. This indicated that an increase in droplet size can effectively reduce droplet drift, which indicated that droplet size is one of the most important factors affecting droplets. Ferguson et al. (2016) applied three different pressures of 207, 310, and 414 kPa to five types of nozzles and found that the number density of droplets is affected by pressure, with higher pressure leading to the largest density. Liu et al. (2021) performed experiments on the liquid distribution performance of a nozzle under four pressures. The experimental results showed that when the pressure was adjusted from 200 to 500 kPa, the value of Dv0.5 ranged from 124.46 to 85.95 μm, and when 400 or 500 kPa liquid was used, the droplets size distribution was more uniform. It can be explained that an increase in pressure can produce more fine droplets and fewer coarse droplets.

Within a certain range (200–500 kPa), the droplet size is affected by pressure. The higher the pressure is, the finer and more uniform the droplet size. Similarly, a smaller droplet size will result in a more serious drift phenomenon, and the larger the droplet is, the worse the penetration. Therefore, it is necessary to select an appropriate pressure level according to different crop canopies to achieve less droplet drift and better droplet penetration.

### Temperature and Humidity

Although the meteorological factors cannot be changed artificially, the influence of temperature and humidity on the deposition of liquid pesticides should be taken into consideration, so as to choose the appropriate ratio of liquid pesticides and water. High temperatures can accelerate the evaporation of droplets and reduce the size of droplets. High humidity causes larger droplet sizes in the air. Low humidity may cause water droplets in the air to shrink due to the diffusion of moisture. With increasing environmental humidity, the size, coverage, and deposition volume of droplets will increase. In the temperature range of 10∼29°C, temperature conditions have no significant effect on droplet deposition (Qi et al., 2020). Franz et al. (1998) determined the relationship between the characteristics of the plant canopy and the weather parameters affecting the aerial spray deposits on cotton and cantaloupe leaves. An increase in relative humidity significantly increased the sediments at the mid canopy level but had no significant effect on the top canopy level. At higher relative humidity, the deposited water droplets tend to be larger. As the leaf area index (LAI) increases, the sediment and size of the water droplets deposited at the mid canopy level decrease. Nuyttens et al. (2005) developed a reliable and feasible spray boom sprayer spray drift measurement program and successfully carried out many drift experiments. These measurements prove the important influence of weather conditions on the drift of deposited spray. A drift prediction equation of a reference spray was established to predict the expected amplitude of the deposition drift under various drift distances and atmospheric conditions (wind speed and temperature). In addition, Tian et al. (2020) has found that the droplet deposition effect and permeability of UAV operations at night are higher than those during the day.

When using UAVs, it is necessary to determine the ratio of pesticides to water and appropriate flight parameters according to the weather conditions.

### Flight Routines in Response to Ambient Wind

Although the magnitude and direction of the ambient wind speed cannot be changed, the adverse effects of ambient wind on the deposition of droplets can be mitigated by adjusting the movement trajectory of the UAV. By quantifying and analyzing the two-dimensional deposition pattern of the UAV, when spraying a single plant, the degree of downwind displacement has nothing to do with the wind speed indicated when the UAV is flying. In the downwind direction, the drift of water droplets is reduced (Li et al., 2019; Richardson et al., 2020). Only in the case of crosswinds will droplet drift occur (Zhang et al., 2015; Fengbo et al., 2017; Shengde et al., 2017a). When the crosswind speed is greater than 3 m/s, the penetrable area is greatly reduced, which is not suitable for operation (Wang L. et al., 2021). With the increase in real-time wind speed and UAV operation altitude, the area within the spray zone and spray deposition have changed significantly. When spraying on pineapple plants, the operating height of the UAV should be below 2.5 m, and the wind speed should be 5 m/s or less (Wang J. et al., 2018). When the parameters of a UAV are a 1.5–3 m flying height and a 2.4–5 m/s flying speed droplet drift only occurs in the downwind direction (Wang X. et al., 2017). As the altitude increases, the drifting resistance of the UAV’s downwash airflow gradually increases (Wu et al., 2019). Hu et al. (2020) used the DQN and PSO algorithms to adjust the flight trajectory according to the environmental wind conditions so that the drift of the droplets was reduced by 50%, and the amount of deposited droplets was increased. Faiçal et al. (2017) used Adaptation to the Environment (AdEn), a system that adjusts the flight according to different wind levels. The experimental results showed that it has better anti-drift performance under system control.

In summary, although we know that when performing plant protection operations in the direction of the environmental wind, the phenomenon of droplet drift is reduced, and the direction of the environmental wind changes all the time, changing the flight direction at all times during the execution of the flight is bound to cause a serious reduction in operational efficiency, therefore more research is needed to improve this aspect.

### Flight Strategy According to Terrain or Plant Shape

In plant protection operations, when facing plants of different shapes, applying different spraying parameters will change the deposition effect. Meng et al. (2020) in order to study the effect of peach tree shape on UAV spray coverage. He divided peach trees into two types: CL shape and Y shape, and concluded that the spraying uniformity of Y and CL types is different. For Y-shaped peaches, the droplet coverage of the outer layer is significantly higher than that of the lower layer, and for CL-shaped peaches, the droplet coverage of the top layer is significantly higher than that of the lower three layers. For Y-shaped peach, “one spray” is preferable to “two sprays” for a given amount of spray per unit area. For CL-shaped peaches, increasing the total nozzle flow from 1.8 to 2.2 L min^–1^ significantly improved droplet coverage at specific locations including the top and bottom two layers. During UAV spraying operations, the ground may be undulating, so the height of the UAV relative to the plant surface may change. Wu et al. (2018) proposed a ground-imitation flight method. The slope is judged by the front-mounted millimeter-wave radar. When the slope fluctuation is small, the differential GPS height and the ground-to-ground millimeter-wave radar height are combined with Kalman filtering to improve the accuracy. When the threshold is reached, the heights of the front-mounted millimeter-wave radar and the ground millimeter-wave radar are fused with multi-radar height information to improve the response speed. Finally, the fuzzy PID control algorithm is used to control the height of the UAV. Through the simulation and field flight test, the goal of the plant protection UAV’s sloping ground imitation flight height error is less than 40 cm.

## Techniques for Improving the Deposition Effect

In addition to changing the UAV operating parameters mentioned in the previous chapter, electrostatic spray, and variable spray techniques can also improve the deposition effect, but different from changing the UAV operating parameters, both techniques require structural adjustments to the spray system, and new control systems must be added. Commonly used spraying techniques were electrostatic spraying and variable spraying. The advantage of electrostatic spraying was that it provided an electrostatic force between the plant surface and the droplets, which actively increases the amount of droplet deposition. The disadvantage was that it was difficult to keep the field strength on the UAV at zero, and the charged droplets may accumulate on the UAV and affect the safety of flight. Variable spray refers to changing spray parameters according to environmental information obtained by sensors, such as wind speed, vegetation height, vegetation shape, etc., thereby actively reducing droplet drift or evaporation. Variable spray mainly changes the spray volume, which does reduce droplet loss, but is not as effective in enhancing droplet deposition as electrostatic spray.

### Improvement of Nozzles

The drift and distribution of droplets depend on the airflow distribution characteristics of the UAV and the droplet size of the nozzle, which is directly related to the control effect of pesticides (Kumar et al., 2018; Sarghini et al., 2019). For spray droplets of different droplet sizes, droplets with small size more easily drift (Wang X. et al., 2018; Yao et al., 2021).

The nozzle is an important part of a plant protection UAV, and its atomization performance directly affects the deposition and drift of pesticides. Commonly used nozzle types are hydraulic nozzles and rotary sprayers. Hydraulic nozzles have a long history and a wide range of types. Wang C. et al. (2021) tested the performance of an IDK120–015 air-injector nozzle and a TR 80–0067 hollow cone nozzle. At a speed of 3.11–3.79 m/s in a crosswind, compared with the traditional HCN nozzles of UAV sprayers, the application of AIN promotes spray deposition and uniformity and significantly reduces deposition and air drift. Kim et al. (2021) proposed a new type of nozzle with a feedback channel, which can spray smaller droplets more uniformly. The existence of the feedback channel effectively reduces the droplet diameter and improves the spray uniformity. Bayat and Bozdogan (2005) combined an air-assisted system with a high-speed rotating disc nozzle to increase spray penetration and deposition and reduce the impact of drift.

The drift potential of commonly used agricultural UAVs is very fragile, The penetration and uniformity of UAVs need to be further improved (Wang et al., 2019b), so it is necessary to actively adopt methods to assist in reducing drift (Xiongkui et al., 2017), such as the tilt of the nozzle angle (Yafei et al., 2019; Yu et al., 2021). The improvement of the nozzle can generally directly affect the spray quality. When improving the nozzle, the balance between uniformity and penetration needs to be considered. At the same time, durability is also an important factor for agricultural nozzles.

### Electrostatic Spray Systems

An electrostatic spray technique refers to the establishment of an electric field between the spray system and the surface of the crop (Chen S. D. et al., 2021), so that the droplets move toward the surface of the crop under the force of an electric field, thereby increasing droplet deposition and penetration ability (Martin and Latheef, 2017; Zhang et al., 2017; Wang S. L. et al., 2018). There are three methods for electrostatic system: the corona charging method, induction charging method, and contact charging method (Law, 2001). The indicator to measure the quality of electrostatic spray is the charge-to-mass ratio (CMR), normally the greater the charge-to-mass ratio, the better deposition effect (Kirk et al., 2001). UAV electrostatic spray systems generally adopt a bipolar connection method to ensure that the UAV is at zero potential (Ru et al., 2015).

In the process of electrostatic spraying by induction charging, the charging electrode directly affects the droplet charging result. With a charge voltage of 8 kV for copper, the charge-to-mass ratio of the spraying system can reach 0.22 mC/kg (Lan et al., 2018). A nickel electrode reaches 1.65 mC/kg at a charging voltage of 3.0 Kv (Patel et al., 2013). Based on the relationship between the conductivity of the liquid, the position of the electrode on the nozzle, and the CMR, a housing for mounting electrodes is designed (Yanliang et al., 2017). Patel et al. (2015) established a CFD model to study the electric distribution and movement of charged droplets, and the results showed that the radial droplet drift has increased with increasing voltage, with smaller droplets drifting more seriously. Al-Mamury et al. (2014) established a COSOML simulation model to analyze the force of the droplets ejected by an electrostatic spray system. These studies have established the foundation for the development of new electrostatic spray systems. An electrostatic pesticide spraying system has been developed that has an outer ring-shaped induction charging electrode around a high-flow hydraulic nozzle (Yamane and Miyazaki, 2008). When the voltage applied to the electrode gradually was increased to 4 kV, the CMR of the spray droplets increased. As the gap between the spray cone and the electrode decreases, the CMR increases. On the other hand, as the nozzle flow rate increases, the CMR decreases.

Compared with traditional spray systems, electrostatic spray can significantly increase the droplet deposition rate (Yang et al., 2018). Martin et al. (2019) used a bipolar electrostatic aerial spray system to spray on early-season cotton at a spray rate of 9.35 L/ha. At a charging voltage of ± 9.0 kV, the electrostatically charged spray significantly increased the deposits on an artificial collector by 34.5% compared with the uncharged spray. Wang S. L. et al. (2018) designed a bipolar contact type aviation airborne electrostatic spray system. Compared with a non-electrostatic spray, electrostatic spray increased droplet deposition by 0.0143 μg/cm^2^. Zhao et al. (2020) proposed a high-voltage electrostatic generator, which is positively and negatively charged for the liquid in two isolated water tanks. A charge transfer circuit was developed in the space between the airborne electrostatic spray system and the ground. This method greatly enhanced the adsorption performance under outdoor conditions. The droplet density on the front of the target has increased by 16.7%.

### Variable Spray Systems and Monitoring Systems

To reduce pesticide waste, a UAV control system needs to execute different spraying parameters according to different environmental conditions (Chen H. B. et al., 2021). Generally, different spray states can be obtained by adjusting the spray switch, flow rate, pressure, and other parameters. This task places high requirements on the accuracy and speed of UAV adjustment. Ling et al. (2018) studied the changing trend of droplet deposition distribution with wind speed, droplet size, flying height, rotor wind direction, and nozzle spray angle under variable spray conditions within a horizontal distance of 1∼4 m, and obtained the variances in the multivariate linear prediction model. Using the basis for real-time control of UAVs, Alves Martins et al. (2020) guessed that vegetation indices (VIs) could be used to estimate application rates for cotton. To maximize cotton yield, a VI based equation that will indicate the ideal application rate was developed. It is concluded that rising vegetation index required increased spray application rates to maintain relative spray deposition in the middle layer of cotton plants. Three different methods for assessing tree row volume (TRV) in ultra-high-density olive orchards using drone photogrammetry were compared by Anifantis et al. (2019). Thus, UAV aerial photography can accurately estimate the amount of biomass in the field, thus giving more accurate prescription maps and further reducing the use of pesticides. Chen et al. (2012) scans the tree’s appearance, height and width, and its leaf density with high-speed laser scanning sensors, and then controls the flow of spraying through solenoid valves. Experiments show that the system has no difference in droplet coverage in the canopy when faced with trees of different sizes and leaf densities. To ensure a uniform spraying amount per unit area, Liu et al. (2020) proposed a set of UAV aerial variable spraying control models and developed a corresponding control system based on the aerial variable spraying technology. The system can adjust an opening of a solenoid valve through a pneumatic variable spray control model according to changes in the flight parameters. The system deviation range is between 0.11 and 9.79%. Wang L. H. et al. (2016) and Cen et al. (2019) used a wind pressure transmitter to measure the flying speed of the UAV. According to the flight speed, pulse width modulation (PWM) for adaptive variable spraying. At the same time, the actual spray flow was measured through a flow sensor, and the spray flow was adjusted through a PID control algorithm based on a BP neural network. Zhu et al. (2010) used a PWM controller to achieve high-precision spray. Chen et al. (2019) proposed a spray system that can adjust the spray angle and pressure according to the wind speed and its changes to reduce droplet drift. Akkoyun (2019) improved the spray range and reduced the use of pesticides by changing the nozzle distribution on the UAV’s boom.

With the development of machine vision technology, the environmental perception ability of UAVs has been greatly improved (Martinez-Guanter et al., 2020). This allows a UAV to adjust the spray parameters in real-time based on the crop and obtained environmental information. This method is usually implemented in two ways. One is to first obtain a map of the crop and then combine the positioning of the UAV to achieve accurate spraying. Yi et al. (2019) proposed a variable spray rate system. The system takes a picture of the sprayed rice field for the first time, divides it into multiple grids, and generates an index map for adjusting the spray rate. Wen et al. (2018) proposed a variable spray system based on a PID and PWM, using the prescription map from ArcMap software to guide the variable spray system to work according to the prescription map. Wang et al. (2019c) used the Lucy-Richardson algorithm to segment the open space and the vegetation area. When the spraying system is in the open space, the system will stop spraying. Campos et al. (2019, 2021) used a multispectral camera mounted on a UAV to obtain a canopy vitality map of the entire plot. It can convert the tree canopy diagram into a practical prescription diagram and modify the working parameters (pressure) in real-time to follow the prescription map. The other method uses sensors to obtain real-time crop and environmental information and then accurately adjusts the spray parameters. Khan et al. (2021) designed a deep learning method to identify the coriander areas in real-time to achieve precise spraying. Hunter et al. (2020a) used UAVs for weed mapping and specific location management (UAV-IS), spraying weeds at specific locations to reduce the use of pesticides. Ex-Green and Ex-Red methods were also performed for the construction of the rice canopy model (Hong et al., 2020). By judging the coverage of the rice canopy under the UAV in real-time, the working status of the UAV nozzle was adjusted to full spray, half spray, and no spray. The response time of the system was largely reduced to 15.765 ms. Considering the demand for saving chemicals, a normalized difference vegetation index (NDVI) algorithm was created to detect the exact location where chemicals are needed (Basso et al., 2019).

In addition to image processing, some researchers used weather sensors or UAV swarms to work together to achieve better spray results. Faiçal et al. (2014a,b) proposed a particle swarm optimization (PSO)-based method for fine-tuning control rules during pesticide spraying on farmland. By considering the weather conditions reported by a wireless sensor network (WSN), this method can be adopted quickly and efficiently. In this case, the UAV becomes the mobile node of the WSN and can make personalized decisions for each farmland. Ivić et al. (2019) proposed an autonomous UAV swarm spraying algorithm control method based on the multiagent area coverage method, thermal equation driven area coverage (HEDAC). Compared with traditional path planning, HEDAC spraying can usually reduce overspray by approximately 3–8%. In practical applications, a UAV spraying group controlled with HEDAC is expected to be significantly better than the UAVs operating using existing path planning methods.

Generally, a variable spray system first needs an excellent algorithm to ensure its adjustment speed and accuracy, and second, it needs to use other sensors to sense the external environment and make decisions that are conducive to pesticide conservation.

## Discussion

The pesticide spraying efficiency of UAVs was closely related to flight parameters including speed, altitude, and pressure. Choosing appropriate flight parameters according to different mission scenarios can effectively improve the spraying effect and reduce pesticide waste. There were three main external factors that affect the spraying effect of drones, environmental wind, terrain flatness and crop height. The improvement of the nozzle improved the uniformity of the spray, and the use of an electrostatic sprayer improves the deposition effect of the pesticide. The use of advanced nozzles coupled with electrostatic spray technology has a very good effect on reducing droplet loss. When working on flat, regular fields, the UAV spray system only needs the electrostatic force of the electrostatic spray system to achieve good deposition results. Variable spray is one of the most effective methods of pesticide utilization under the current technological capabilities. Variable spraying is especially important in rugged, irregular fields, or when plants vary in height and shape. Through continuous integration of new technologies and new algorithms, UAVs equipped with variable spray systems will have a better environmental perception, path decision-making capabilities, and spray control accuracy.

In addition, it is also an idea to reduce pesticide waste by introducing other machines (Lan and Chen, 2018) or chemicals. Liu et al. (2019) uses a combination of UAVs and motorboats. The UAV provides GPS positioning and the motorboat is used for weeding in paddy fields, avoiding the problem of pesticides drifting in the air. A pre-wetting process can reduce the contact angle between the droplet and the blade, making it easier for the droplet to wet and spread, thereby increasing the amount of deposition and improving efficiency (Yao et al., 2018). Dicamba hydrogel was added to the chemical solution to increase the deposition of the chemical solution and reduce the environmental pollution caused by the drift of pesticide spray (Song et al., 2021).

## Conclusion and Prospect

**FIGURE 2:**
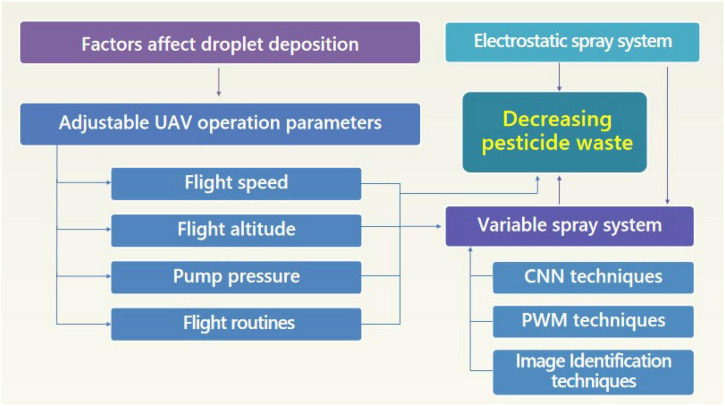
The relationship network of various methods and their reduction in pesticide waste.

The above references first introduced the factors that affect the deposition of pesticides, including the UAV flight parameters and environmental parameters. Second, to cope with the above factors and reduce pesticide waste, the improvement of the spray nozzles and spray principles made by the researchers, and the proposal of different variable spray algorithms are introduced. Their relationship is shown in Figure 2. In the actual design of a UAV and spray systems to reduce pesticide waste, it may be necessary to adjust various parameters and combine several techniques. In addition, due to different references using different UAVs and spray systems, those references may become inapplicable, especially when introducing new techniques Using an existing UAV system, when setting the flight parameters, a speed of 1.5–3 m/s, and a height of 2–3.5 m based on different crop canopies, can achieve relatively good spray uniformity. To further improve the utilization rate and spray effect of pesticides, it is necessary to improve the UAV system or propose a new control algorithm. Compared with improving the UAV hardware, it is very cost-effective to propose new algorithms to better control the UAV spray. Two possible directions that have not been studied have been discovered. First, when the UAV is accelerating, it will inevitably have a forward tilt attitude. This phenomenon is the principle of a UAV flying forward, and will cause the spray system to have an inclination angle with the ground. A larger tilt angle resulted in more unfavorable droplet deposition. Despite the research on stabilizing the attitude of the UAVs in flight, a tilt angle of the spray system to the ground still exists. If a mechanism is set up to change the tilt angle of the spray system, so that it is always perpendicular to the ground, the droplet deposition effect will be further improved. In addition, some factors that affect the deposition of droplets, some that have not been studied, so it is impossible to make corresponding adjustments for reducing pesticide waste. By establishing a simulation model and using a neural network, the parameters are studied for more adjustment.

## Author Contributions

HH, HZ, JH, and KF: conceptualization. HH, YK, and JH: methodology. HH, ML, and KI: resources. HH: writing—review and editing. All authors contributed to the article and approved the submitted version.

## Conflict of Interest

The authors declare that the research was conducted in the absence of any commercial or financial relationships that could be construed as a potential conflict of interest.

## Publisher’s Note

All claims expressed in this article are solely those of the authors and do not necessarily represent those of their affiliated organizations, or those of the publisher, the editors and the reviewers. Any product that may be evaluated in this article, or claim that may be made by its manufacturer, is not guaranteed or endorsed by the publisher.
